# Direct Femtosecond Laser Fabrication of Chemically Functionalized Ultra-Black Textures on Silicon for Sensing Applications

**DOI:** 10.3390/nano11020401

**Published:** 2021-02-04

**Authors:** Yulia Borodaenko, Stanislav Gurbatov, Mikhail Tutov, Alexey Zhizhchenko, Sergei A. Kulinich, Aleksandr Kuchmizhak, Aleksandr Mironenko

**Affiliations:** 1 Far Eastern Federal University, 690091 Vladivostok, Russia; serbm@mail.ru (Y.B.); thunderbird87@mail.ru (M.T.); skulinich@tokai-u.jp (S.A.K.); 2 Institute of Automation and Control Processes FEB RAS, 5 Radio St., 690041 Vladivostok, Russia; gurbatov_slava@mail.ru (S.G.); g89leksig@mail.ru (A.Z.); 3 Institute of Chemistry FEB RAS, 159 Pr. 100-let Vladivostoka, 690022 Vladivostok, Russia; 4 Research Institute of Science and Technology, Tokai University, Hiratsuka, Kanagawa 259-1292, Japan

**Keywords:** femtosecond laser pulses, laser ablation in liquids, laser-induced periodic surface structures (LIPSS), surface functionalization, surface enhanced fluorescence (SEF), rhodamine 6G

## Abstract

Here, we present the single-step laser-assisted fabrication of anti-reflective hierarchical surface textures on silicon locally functionalized with a photoluminescent (PL) molecular nanolayer. Using femtosecond-laser ablation of commercial crystalline Si wafers placed under a layer of a solution containing rhodamine 6G (R6G) a triethoxysilyl derivative, we fabricated ordered arrays of microconical protrusions with self-organized nanoscale surface morphology. At the same time, the laser-induced temperature increase facilitated surface activation and local binding of the R6G derivative to the as-fabricated nanotextured surface. The produced dual-scale surface textures showed remarkable broadband (visible to near-IR) light-absorbing properties with an averaged reflectivity of around 1%, and the capping molecular nanolayer demonstrated a strongly enhanced PL yield. By performing a pH sensing test using the produced nanotextured substrate, we confirmed the retention of sensory properties of the molecules attached to the surface and validated the potential applicability of the high-performing liquid-assisted laser processing as a key technology for the development of innovative multifunctional sensing devices in which the textured substrate (e.g., ultra-black semiconductor) plays a dual role as a support and PL signal amplifier.

## 1. Introduction

Surface modification of solids is an effective approach towards the development of multifunctional materials for various biomedical, optical and optoelectronic applications. The concept of surface modification combines a set of processing methods that permit one to change a wide range of physical and chemical characteristics of the modified material, also affecting its wettability [1], corrosion resistance [2], tribological [3], optical [4] and optoelectronic [5] characteristics, etc. Among others, silicon (Si) presents an earth-abundant element, which as a semiconductor that exhibits remarkable optoelectronic and optical properties. All this makes it extremely important for the realization of photodetectors, solar cells, micro-electronic devices and optical sensors [6,7,8,9,10,11,12]. In this respect, the rapidly growing interest in the realization of various devices stimulates the search for efficient and simple approaches for the surface engineering of silicon-based semiconductor materials.

In general, surface modification of Si can be carried out using various physical and chemical methods (and their combinations). Etching and chemical surface functionalization can be classified as chemical approaches to surface processing [13]. For example, functionalization of silicon can proceed either via binding alkoxysilanes to terminal silanol groups of native oxide layer [14], or through the addition of unsaturated hydrocarbons [14,15] and carbonyl compounds [14] to hydrogen-terminated surface. Physical methods include the formation of a certain surface morphology or local modification of chemical composition via doping, implantation or nanopore incorporation [16,17,18]. Among these physical methods, direct laser processing of a silicon surface with nano- and femtosecond (fs) laser pulses is an innovative, convenient, green and high-performing technology for the fabrication of surfaces with various morphologies [18,19,20,21]. In particular, liquid-assisted pulsed laser processing of Si was shown to enable large-scale fabrication of homogeneous nanotextured surfaces exhibiting feature size around 100 nm, i.e., well below the optical diffraction limit [22]. At the same time, ultra-fast deposition of laser energy onto a material creates unique experimental conditions (high local pressures and temperatures [23,24]) allowing the silicon substrate to efficiently interact with surrounding media (liquid or gaseous) via laser-induced chemical reactions. This provides a unique opportunity for patterning a Si surface simultaneously with its functionalization. For example, laser ablation of Si in chemically-active carbon disulfide was found to produce ultrafine surface nanoripples doped with sulfur that absorbed light in the mid-IR spectral range [25,26].

Here, we demonstrate a single-step formation of hierarchical surface textures locally functionalized with photoluminescent (PL) molecular nanolayer upon direct fs-laser processing of commercial Si wafers in a solution containing rhodamine 6G (R6G) triethoxysilyl derivative. By scanning the Si surface with fs-laser pulses along a mesh-like path and keeping a few-micron line interval, we fabricated ordered arrays of microconical protrusions with self-organized nanoscale surface morphology driven by the interference of surface plasmon waves. At the same time, a laser-induced increase in temperature facilitates surface activation and local binding of the R6G derivative to the nanotextured surface “in situ”. The produced dual-scale surface textures show remarkable broadband (visible to near-IR) anti-reflection performance with an averaged reflectivity around 1%, and the capping molecular nanolayer demonstrates a strongly enhanced PL yield. All this makes such functionalized nanotextured Si surfaces promising for optical sensing applications.

## 2. Materials and Methods

### 2.1. Synthesis and Characterization of Functionalizing Compound *(**1**)*

R6G (Sigma-Aldrich, St. Louis, MO, USA, 99%), (3-aminopropyl) triethoxysilane (APTES, Sigma-Aldrich, 99%), ethanol (Sigma-Aldrich, 99%) and Na_2_CO_3_ (Sigma-Aldrich, 99%) were used as received. First, rhodamine 6G (0.5 mmol, 0.24 g) and APTES (0.5 mmol, 0.11 g) were dissolved in ethanol (15 mL), after which finely ground Na_2_CO_3_ (0.2 g) was added to the solution. The mixture was refluxed for 4 h under stirring. Then, the solvent was removed from the reaction mixture at reduced pressure to prepare a product as a red viscous oil (100%), which was then used without further purification.

NMR spectra were measured at the frequency of proton resonance 400 MHz using CDCl_3_ as solvent (Avance 400, Bruker, Billerica, MA, USA). ^1^H NMR (400 MHz, CDCl_3_, ppm, *δ*): 7.95 (m, 1H), 7.47 (m, 2H), 7.08 (m, 1H), 6.35 (s, 2H), 6.29 (s, 2H), 3.51 (br.s, 2H), 3.72 (q, 6H), 3.22 (q, 4H), 2.84 (m, 2H), 1.85–1.92 (m, 6H+2H), 1.32 (t, 6H), 1.25 (t, 9H), 0.66 (br.s, 2H); elemental analysis data (calc. for C_3_5H_4_7N_3_O_5_Si): Calc. C, 68.04; H, 7.67; N, 6.80; Si, 4.55; Expt. C, 67.86; H, 7.71; N, 6.75; Si, 4.49.

### 2.2. Fabrication

**Figure 1:**
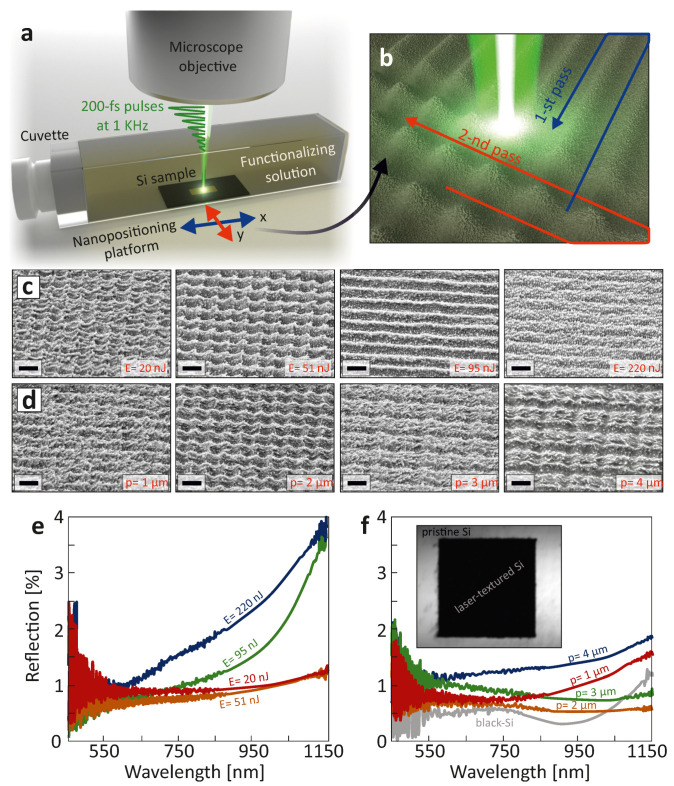
Fabrication of chemically functionalized ultra-black Si surface. (**a**) Artistic representation of the setup for liquid-assisted fs-laser texturing and functionalization of Si surface. (**b**) Illustration of the double-pass scanning scheme to process the Si surface with the laser beam. (**c**,**d**) A series of side-view (view angle of 45°) SEM images of the Si surface processed at varied pulse energies *E* and lateral intervals between scanning lines *p*: (**c**) *E* increases from 20 to 220 nJ at *p* = 2 μm; (**d**) *p* increases from 1 to 4 μm at fixed *E* = 51 nJ. Fixed *κ* = 1 KHz and *ν* = 0.5 mm/s were used. Scale bar indicates 2 μm. (**e**,**f**) FTIR reflection spectra measured from corresponding nanotextured surfaces shown in panels (**c**,**d**). Inset image in (**f**) shows the bright-field optical image of the square-shaped (200 × 200 μm^2^) laser-textured Si surface.

Commercial monocrystalline Si wafers were used as supplied. Laser nanotexturing was carried out with second-harmonic (*λ* = 513 nm) 200-fs linearly polarized laser pulses from a Yb:KGW-based laser system (Pharos, Light Conversion, Vilnius, Lithuania) at a fixed pulse repetition rate of 1 KHz. Laser radiation was focused on the sample surface by a dry microscope objective (Nikon, Tokyo, Japan) with a numerical aperture (NA) of 0.3, yielding in a focal spot size of ≈2 μm. The samples were processed in a cuvette completely filled with 1 g/L of solution 1 in 95% methanol, as schematically illustrated in Figure 1a. The thickness of the liquid layer above the Si surface was less than 1 mm to avoid excessive absorption of laser radiation. The cuvette was arranged onto a motorized PC-driven nanopositioning platform (ANT series, Aerotech GmbH, Nurnberg, Germany) permitting the sample surface to be scanned with the laser beam along the predefined mesh-like trajectory with a constant scanning speed of 0.5 mm/s (Figure 1b). Polarization of the laser radiation was maintained to be oriented along the scanning direction using an adjustable *λ*/2-waveplate. Pulse energy *E* incident to the sample surface was measured with a pyroelectric photodetector (Ophir Optronics, Jerusalem, Israel) and adjusted with a motorized attenuator (Standa, Vilnius, Lithuania). After fabrication, the samples were removed from the solution and cleaned several times with acetone via ultrasonication. For comparative studies, a smooth silicon sample functionalized with the same compound was also prepared. For this, we immersed a pre-activated (via sonication in NH_3_/H_2_O_2_/H_2_O (1/1/1, *v*/*v*/*v*) for 30 min) Si substrate into the functionalizing solution for 48 h to yield a homogeneous organosiloxane functional layer. The thickness of this layer was measured by spectroscopic ellipsometry (Auto SE, Horiba France SAS, Longjumeau, France) to be ≈25 nm.

### 2.3. Characterization

Morphology of the laser-textured Si surface was characterized using scanning electron microscopy (SEM; Ultra 50+, Carl Zeiss, Oberkochen, Germany). Reflectivity of the laser-textured Si was evaluated in the visible and near-infrared (IR) spectral range using a Fourier transform infrared (FTIR) microscope (Hyperion 3000, Bruker, Billerica, MA, USA) coupled to an IR spectrometer (Vertex 80v, Bruker, Billerica, MA, USA). The measurements were carried out in reflection mode with a Cassegrain lens with NA = 0.5 by averaging 500 scans at a spectral resolution of 8 cm^−1^. All spectra were normalized to the signal measured from a smooth silver mirror. For comparison, we also measured reference reflection spectra from pristine Si wafers, and from a “black silicon” (b-Si), a well-known nanotextured substrate with ultra-low reflectivity. Such samples were fabricated using a simple reactive ion etching procedure in O_2_/SF_6_ gas mixture according to the protocol described elsewhere [27,28]. Binding of the functionalizing compound to the laser-textured surfaces was confirmed by measuring mid-IR reflection spectra under vacuum conditions within a spectral range from 2.5 to 25 μm, where molecular vibration fingerprints can be clearly identified. For these measurements, two laser-textured areas with lateral size around 1 × 1 cm^2^ were produced in functionalizing solution and in pure solvent.

PL properties of the chemically functionalized laser-textured Si were studied using a home-built setup comprised of an optical microscope confocally aligned with a grating-type spectrometer (Shamrock 303i, Andor Technologies, Belfast, Northern Ireland) equipped with a thermoelectrically cooled CCD-camera (Andor Technologies, Belfast, Northern Ireland). Linearly polarized laser radiation generated by a CW semiconductor laser (Melles Griot, Rochester, NY, USA) with a wavelength of 532 nm hit the sample surface from the top using a dry microscope objective with NA = 0.15 (Nikon, Tokyo, Japan). The same objective was used to collect the signal that was further analyzed either with a spectrometer or with an additional CCD-camera (Nikon, Tokyo, Japan) capturing PL images through an appropriate Notch filter. The size of the pump laser spot in the image plane was adjusted by a beam expander, while the signal acquisition area for spectroscopic measurements was tailored by varying the diameter of optical fiber connecting the spectrometer and the optical setup. The pump laser intensity (≈10 mW/cm^2^) was chosen to avoid photodegradation of the PL signal that remained stable for at least 1 min of continuous exposure. When measuring PL of samples in a dry state, they were pre-treated with 1 M HCl solution and air dried to activate PL properties. The optical setup was also used to measure the variation of PL signal from the textured surface capped with solutions with varied pH that was controlled by pH meter. The solutions were obtained by successive dilution of 1 M HCl with deionized water. The measurements were performed with a home-built liquid cell.

## 3. Results and Discussion

### 3.1. Femtosecond-Laser Liquid-Assisted Processing of Silicon

Several previous studies reported fs-laser ablation of Si wafers in liquid environments (including water, ethanol and acetone), where multiple laser processing parameters such as pulse energy *E* (fluence, *F*), pulse repetition *κ* and scanning rate *ν* (or number of applied pulses per spot) were shown to affect the resulting surface morphology via multiple physical phenomena proceeding upon liquid-assisted laser ablation [22,29,30,31,32]. With no loss in generality, to illustrate the key idea of this paper, here we considered Si processing at *κ* = 1 KHz and *ν* = 0.5 mm/s (i.e., practically relevant parameters available for majority of commercial fs lasers and nanopositioning/scanning systems), and only the pulse energy *E* and the lateral interval between laser scanning lines *p* were adjusted. In particular, each surface area with an overall lateral size of 200 × 200 μm^2^ was first processed following a snake-like trajectory along one direction; and then, the textured area was scanned again along the orthogonal direction while preserving the same *p* (Figure 1b).

Variation of the above-mentioned laser processing parameters (*E* and *p*) allowed us to produce diverse surface morphologies on the Si surface upon its laser-induced melting and ablation in the presence of functionalizing solution. The series of SEM images in Figure 1c illustrates how the surface morphology evolves upon increasing pulse energy from 20 to 220 nJ while keeping the same interval between scanning lines *p* = 2 μm. At moderate pulse energies, the images reveal formation of hierarchical surface morphology, with microconical protrusions with nanoscale roughness arranged at a period equal to *p*. At higher *E*, the surface topography first changed to an ordinary array of trenches whose orientation and periodicity coincide with the direction of the second pass of laser beam (Figure 1b). This indicates that upon the second pass of the laser beam along the orthogonal direction, the already formed surface textures were completely erased. Then, even such trenches almost disappeared at even higher pulse energies, while high-spatial frequency LIPSS with the characteristic period ≈130 ± 15 nm were observed (right-most image in Figure 1c). Such a behavior could be indicative of defocusing of the incident laser beam via filamentation in liquid. For a fixed pulse repetition rate (*κ* = 1 KHz), larger beam size was found to increase spatial overlap between adjacent pulses on the Si surface, which facilitated LIPSS formation through the improved positive feedback [33,34]. The orientation of formed LIPSS was found to be perpendicular with respect to the polarization vector of the second-pass laser beam. This suggests interference of surface plasmon waves on the surface of photo-excited Si as a mechanism driving such nanoscale self-organization, in accordance with the previous studies [35]. Noteworthily, a further increase in pulse energy leads to formation of vapor bubbles that strongly deteriorate laser beam focusing on the sample surface and thus result in nonuniform surface texturing.

Figure 1d demonstrates how the surface morphologies of processed samples change at fixed *E* = 51 nJ and different values of lateral interval between scanning lines *p* varied from 1 to 4 μm. As clearly seen, the hierarchical micro- and nanoscale surface morphology cannot be imprinted when *p* is smaller than the focal beam size, even at pulse energies below the filamentation threshold, owing to erasure of the main trenches after the second pass of the laser beam. Indeed, the microscale morphology can be potentially imprinted at shorter periods when high-NA optics is used to focus laser beam. However, such tight focusing also reduces focal depth of the laser spot, thereby complicating surface alignment when patterning large surface areas. From the practical application point of view, even larger (≈5–10 μm) laser spots supported by commercial galvanometric scanning systems should be considered. In any case, further optimization of laser processing parameters is not seen to be within the scope of this report.

The fabricated surface textures were found to demonstrate remarkable morphology-dependent anti-reflecting properties. In particular, the reflection of the pristine Si wafer in the visible and near-IR spectral range is seen in Figure 1e,f to drop from ≈35% to around 1–2.5% upon its texturing with fs-laser in the functionalizing solution. The hierarchical surface morphologies combining microconical protrusions with nanoscale features produced at moderate pulse energies *E* reached averaged reflectivity values as low as 1%, which is comparable with those for black-Si (gray curve in Figure 1f), the latter material being a benchmark for ultra-black surfaces. Low reflectivity of the laser-textured Si originates from the enhanced light absorption by the multi-scale surface features. Analysis of the data presented in Figure 1e,f reveals the importance of hierarchical morphology for achieving broadband absorption. For example, reflection from surface textures with shallow height modulation (nanoripples) gradually increases with wavelength. In addition, the gradual change of the refractive index at the air–Si interface also contributes to the light-absorbing performance. In particular, densely arranged nanoscale features can be considered as a surface layer with a lower (higher) effective refractive index than that of silicon (air). This decreases the refractive index jump at the interface, reducing the overall Fresnel reflection, allowing the pump radiation to couple the surface more efficiently [36].

### 3.2. Chemical Functionalization and pH Sensing

**Figure 2:**
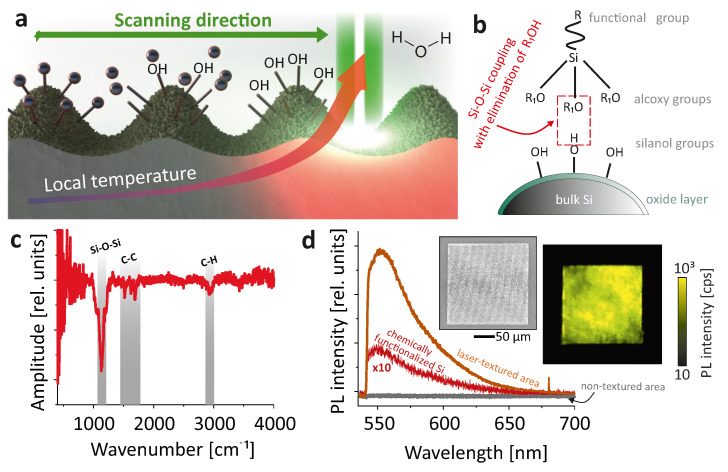
(**a**) Scheme of surface functionalization during fs-laser processing. (**b**) Scheme of surface chemical reaction. (**c**) Normalized FTIR reflection spectrum of the functionalized laser-textured Si surface. (**d**) Averaged PL spectra measured from the laser-textured Si (orange line), non-textured surrounding area (gray line) and chemically functionalized smooth Si (red line). Insets show reference SEM and PL images of a 200 × 200 μm^2^ laser-textured area.

As mentioned above, functionalization of silica surfaces is a well-developed technology combining many chemical approaches. Since under normal conditions, bulk Si is coated with a thin oxide layer (which is formed by oxygen, moisture or other chemicals), this technology is also used to functionalize Si surfaces. When interacting with a high-energy laser pulse, local temperatures reach extremely high values, allowing silicon to actively interact with water. In our experiments, 95% methyl alcohol was used as a solvent; thus, after laser processing the surface immediately interacted with water to form surface silanol groups (Si–OH) as schematically illustrated in Figure 2a. Then, at still relatively high temperatures, these surface groups condensed with alkoxy (Et–O–Si) and hydroxy (HO–Si) groups of functionalizing compound to form Si–O–Si bonds via either alcohol or water elimination, as shown in Figure 2b.

Despite the weak signal due to the small thickness of the grafted functional layer, the FTIR spectrum (Figure 2c) can clearly distinguish several characteristic bands: Si–O–Si vibration at 1100 cm^−1^, and aromatic ring C–C and C–H stretching vibrations at 1500–1700 cm^−1^ and 3000 cm^−1^, respectively. Upon photoexcitation with laser (532 nm), the textured area exhibited intense PL with a maximum at 557 nm (Figure 2d, orange spectrum), while the surrounding surface area remained dark (Figure 2d, gray spectrum). For comparison, we measured a PL signal from a smooth Si sample chemically functionalized with a uniform organosiloxane layer with a thickness of ≈25 nm (see Materials and Methods), which demonstrated 30-fold less intense PL intensity compared with the laser-recorded structure (Figure 2d, red spectrum). All these findings confirm the hypothesized mechanism of surface functionalization “in situ”—i.e., in the process of fs-laser ablation, and possible applicability of the presented method for the development of multifunctional materials with controlled optical and chemical properties.

Covalent bonding of sensory compounds to substrates is a popular sensor design strategy for solution analysis. In contrast to encapsulation, imprinting and adsorption, it provides easier access for the analyte to the sensitive receptors, also preventing leaching of the sensory compound. The thickness of attached layers is usually on the order of several nanometers, which requires sufficiently sensitive equipment when optical chemosensors are used. In this work, a thin fluorescent layer was attached to a nano-textured substrate, which, as was shown earlier [34,37], provides a significant enhancement of PL emission that can be easily detected by standard equipment. Noteworthily, dielectric structures are advantageous for the realization of the sensors based on PL enhancement effects. In particular, such nanostructures do not suffer from fluorescence quenching and passively cancel electron-driven catalytic reactions, in a sharp contrast to plasmonic nanostructures [28]. Additionally, structures made of dielectric low-loss semiconductors with high refractive index supports enhanced electromagnetic near-fields at the interface via light trapping and excitation of Mie-type resonances that facilitates PL excitation and emission [9,10].

**Figure 3:**
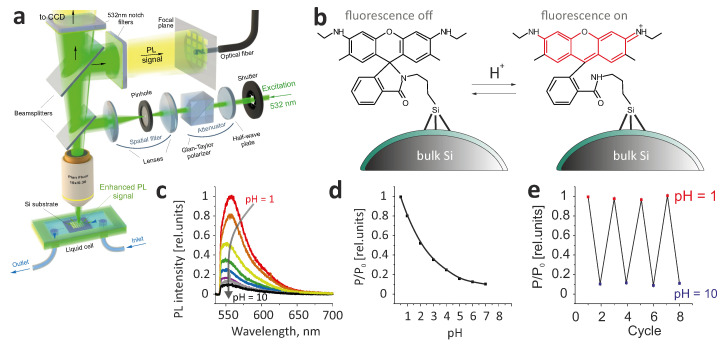
(**a**) Experimental setup for PL measurements and pH sensing tests. (**b**) Spirolactam ring-opening reaction. (**c**) A series of PL spectra of the pH sensitive laser-textured surface upon successive acidification of the surrounding solution. (**d**) Dependence of the normalized PL intensity P/P_0_ on pH value. (**e**) Change in signal intensity upon cyclic filling of the microfluidic cell with basic (pH = 10) and acidic (pH = 1) solutions.

The compound used for functionalization, R6G spirolactam derivative, is known to display a red color change and strong PL in acidic solution by activating a carbonyl group in its spirolactam moiety [38,39], as shown in Figure 3b. Figure 3c,d shows how PL spectra change upon acidification of the solution placed atop of a laser-textured functionalized sample and the normalized PL intensity P/P_0_ (where P_0_ is PL intensity at pH = 1) versus pH value (see Materials and Methods for details). According to these data, binding of the functionalizing compound to the surface does not significantly affect its pH-sensitive response, and repeated filling of the microfluidic cell by solutions with different pH values did not show any significant degradation of the sample due to leaching of the functionalizing compound, as clearly seen in Figure 3e).

## 4. Conclusions

In this study, direct fs-laser processing was performed to produce light-absorbing surface textures on silicon locally functionalized with a photoluminescent R6G nanolayer. Microscale laser ablation accompanied by nanoscale plasmon-mediated self-organization allowed us to create hierarchical surface morphologies possessing remarkable light-absorbing properties (average reflectivity around 1%). Additionally, the surface textures boosted the PL signal from the capping R6G nanolayer that can be used to realize various sensing devices.

We used reflectivity of the visible-light radiation as the simplest benchmark of the surface-enhanced photoluminescent effect [40], i.e., the ability of the textures to enhance the R6G PL signal. However, multiple effects, such as optimized surface area and near-field localization and enhancement of the electromagnetic fields by nanoscale surface features should be taken into account and optimized to achieve maximal PL yield upon low-intense, non-destructive optical excitation of the sensing nanolayer, which is relevant for stable sensor operation and maximal sensitivity. In sharp contrast to reactive ion etching of Si wafers, direct fs-laser fabrication of “black” Si represents a highly precise maskless procedure that can be used to realize arbitrary design of the sensor element. From this point of view, the technology seems especially promising for the fabrication of sensor arrays, where each element can be functionalized with a certain analyte, for multi-component real-time monitoring of water systems. Additionally, the presented fabrication technique can be easily integrated with an existing planar process for the production of silicon integrated circuit chips, and should be considered as a viable approach toward the development of so-called lab-on-chip sensing devices.

Noteworthily, the demonstrated pH sensing represents the simplest application of spiroring-opening reaction and was chosen only to demonstrate the key idea of the work. Generally, spironolactams and spironolactones of xanthene dyes, especially rhodamines and fluoresceins, are well-known platforms for the development of selective chemosensors, since the introduction of a selective cation-binding ligand into the spiro ring system imparts selectivity to the sensory response [38,39,41,42]. This means that selective cation-sensitive surfaces can be realized with a proper chemical design of the functionalizing compound.
